# Emergence of psychiatric adverse events during antipsychotic treatment in AP-naïve children and adolescents

**DOI:** 10.1186/s13034-022-00517-3

**Published:** 2022-11-12

**Authors:** Marie-Line Menard, Philippe Auby, Coralie Cruzel, David Cohen, Olivier Bonnot, Florence Askenazy, Susanne Thümmler, ETAPE Study Group

**Affiliations:** 1 Department of Child and Adolescent Psychiatry, University, Children’s Hospitals of Nice CHU- Lenval, 57 avenue de la Californie, 06200 Nice, France; 2 CoBTek, EA7276, Université Côte d’Azur, Nice, France; 3 Department of Clinical Research and Innovation (DRCI), Nice University Hospital, Nice, France; 4 Department of Child and Adolescent Psychiatry, GH Pitié-Salpêtrière, APHP, Paris, France; 5 CNRS UMR 7222 Institut des Systèmes Intelligents et Robotiques, Pierre & Marie Curie University, Paris, France; 6 Department of Child and Adolescent Psychiatry, University Hospital, Nantes, France

**Keywords:** Antipsychotic, Children, Adolescents, Psychiatric adverse events

## Abstract

**Background:**

Over the last decades, antipsychotic prescriptions in children have increased worldwide. However, adverse events are frequently observed, with some such as psychiatric adverse events remaining poorly documented.

**Method:**

The French ETAPE study is a 12-month naturalistic prospective multisite study that included 190 antipsychotic-naïve pediatric patients (mean age = 12 ± 3 years), treated by antipsychotic for psychotic or non-psychotic symptoms. From the ETAPE database, we performed additional analyses focusing on psychiatric adverse events.

**Results:**

Children received mainly second-generation antipsychotic for conditions out of regulatory approval, with risperidone and aripiprazole being the most frequent (respectively 52.5% and 30.83%). Clinicians reported 2447 adverse events, mainly non-psychiatric (n = 2073, 84.72%), including neuromuscular, metabolic, gastroenterological, and (n = 374, 15.28%) psychiatric. 55.88% of psychiatric adverse events were attributable to antipsychotic by the clinician, compared to 89% of non-psychiatric adverse events (*p < 0.001*). 63.2% (n = 120) of the 190 children and adolescents presented at least one psychiatric adverse event. The most frequent were externalized behaviors such as aggressiveness or agitation (22.7%), mood changes (18.4%) and suicidal ideas or behaviors (11.8%). Half of psychiatric adverse events occurred during the first quarter, 49.46%, compared to 23.79% during the second, 15.77% during the third, and 10.96% during the fourth.

**Conclusion:**

This additional analysis from the French ETAPE study emphasizes that psychiatric adverse events might be more frequent than expected in the pediatric population. Also, the potential risk of psychiatric adverse events should be part of the benefit-risk evaluation and sub-sequent follow-up.

**Supplementary Information:**

The online version contains supplementary material available at 10.1186/s13034-022-00517-3.

## Background

All over the world, the use of antipsychotics (AP) is widespread in the pediatric population for psychotic and non-psychotic disorders [1–4]. Among AP, second-generation AP such as risperidone and aripiprazole are the most widely used in the pediatric population [5]. Their regulatory approval for some psychiatric indications (e.g. by the Food and Drug Administration or the European Medicines Agency) in the pediatric population and their better neuromuscular tolerance than first-generation AP explain their predominant use [6]. However, in current practice, AP prescriptions go far beyond the framework of agencies’ approvals [7, 8].

In light of the high frequency of AEs reported in the literature, the frequent use of APs remains a concern [9]. These AEs can worsen the morbidity of young patients (e.g. weight gain, metabolic syndrome, hormonal). Furthermore, specific AE-profiles, according to different AP molecules, are reported [9–14]. Guidelines are available worldwide (e.g. AACAP in the United States, CAMESA in Canada or NICE in the United Kingdom) and provide guidance to healthcare professionals on how to monitor the use of AEs with children treated with APs [15]. So for these common AEs, the prescriber is sensitized to the need for regular and repeated monitoring, including clinical and paraclinical parameters throughout exposure to APs [16–18]. Even so, the literature points out a low level of adherence to follow-up guidelines concerning AP treatments in the pediatric population [19, 20].

However, several AEs remain poorly documented in AP-naïve pediatric patients [21], including psychiatric AEs [3, 22–24]. The French ETAPE study is a 12-month naturalistic prospective multisite study that included 190 AP-naïve pediatric patients (mean age = 12 ± 3 years), treated by AP for psychotic or non-psychotic symptoms. Here, we performed additional analyses from the French ETAPE study database focusing on psychiatric AEs.

## Method and statistical analysis

ETAPE is a French multisite, naturalistic and observational study aiming to determine the incidence of AEs potentially attributed to AP treatment prescribed for psychotic or non-psychotic symptoms in AP-naïve pediatric patients. Standard definitions and terminology for key aspects of clinical safety reporting are taken from the Clinical Safety Data Management of the European Medecines Agency [25]. Our research used the Pediatric Adverse Events Rating Scale (PAERS) to systematically search and identify AEs over a 12-month follow-up period with four quarterly visits : at the end of the first quarter (Q1), the second (Q2), the third (Q3), and the fourth (Q4). We classified AEs into two clinical dimensions: (1) psychiatric AEs including aggressivness/agitation/challenging behaviors, mood changes, suicidal ideation/behavior, apathy/restricted range of emotion/lack of interest, irritability, trouble paying attention/concentrating, anxiety, hallucinations, racing thoughts, sexual dysfunction, psychiatric relapse; and (2) non-psychiatric AEs including neuromotor, metabolic, gastroenterological, eating, hormonal, sleep disorders, dermatologic, hematologic, cardiologic [26]. The on-site investigator assessed the causality [27]. For each AE, the clinician in charge of the patient determined if it was attributable to the AP drug (probably attributable, possibly attributable or non attributable) and ranked the severity of the AE (mild, moderate, severe or extreme) based on his or her expertise. We also monitored other parameters: anthropometrics measures, blood pression, blood tests and electrocardiogram. The ETAPE protocol and the main results have been presented previously [26, 28].

This manuscript describes the additional analysis of the French ETAPE study data focusing on psychiatric AEs. Patient characteristics entering the study are presented in Table 1, including age, sex, Tanner status, clinical diagnosis, global severity with the Clinical Global Impressions-Scale (CGI-S) and the Children Global Assessment Scale (CGAS), and the AP drug prescribed. Table 2 reports psychiatric AEs.

**Table 1:** Characteristics of the population

Variables	Total ETAPE sample	Group of patients with at least one psychiatric AE		
		**All psychiatric AEs**	***AEs Attributable*** ***to AP***	***AEs*** ***Non attributable*** ***to AP***
**N** % (n)	100% (190)	63% (120)	75% (*95)*	25% (*25)*
**Age mean** (± SD)	12.1 (**±** 2.9)	12.2 (**±** 3.1)	*12.3 (***±** *3.1)*	*11.9 (***±** *2.8)*
**Sex** % (n)				
Boys	73.7% (140)	70.8% (85)	*72.6% (69)*	*64% (16)*
Girls	26.3% (50)	29.2% (35)	*27.4% (26)*	*36% (9)*
**Tanner status** % (n)				
I Prepuberty	32.6% (62)	33.3% (40)	*31.6% (30)*	*40% (10)*
II-IV Puberty in progress	42.7% (81)	40% (48)	*41% (39)*	*36% (9)*
V Puberty completed	24.7% (47)	26.6% (32)	*27.4% (26)*	*24% (6)*
**Clinical diagnoses (DSM)** % (n)				
Schizophrenia spectrum and other psychotic disorders	27.9% (53)	30% (36)	*29.5% (28)*	*32% (8)*
Disruptive, impulse-control, and conduct disorders	19.5% (37)	19.2% (23)	*20% (19)*	*16% (4)*
Bipolar disorders	11% (21)	9.2% (11)	*7.4% (7)*	*16% (4)*
Autism spectrum disorder	10.5% (20)	10.8% (13)	*11.6% (11)*	*8% (2)*
Personality disorders	9.5% (18)	10.8% (13)	*12.6% (12)*	*4% (1)*
Anxiety disorders	7.4% (14)	9.2% (11)	*8.4% (8)*	*12% (3)*
Eating disorders	4.7% (9)	4.2% (5)	*4.2% (4)*	*0*
ADHD	3.1% (6)	3.3% (4)	*3.1% (3)*	*4% (1)*
Tourette syndrome	2.6% (5)	2.5% (3)	*2.1% (2)*	*0*
Fragile X syndrome	0.5% (1)	0.8% (1)	*1% (1)*	*0*
Others	3.1% (6)	0	*0*	*0*
**CGI-S mean** (± SD)	4.83 (**±** 0.84)	4.85 (**±** 0.92)	*4.82 (*± *0.92)*	*4.96 (*± *0.91)*
**CGAS mean** (± SD)	47 (**±** 10.7)	46.2 (**±** 9.9)	*45.6 (*± *9.3)*	*48.3 (*± *12)*
**AP drug**				
Risperidone	50.5% (96)	52.5% (63)	*54.7% (52)*	*44% (11)*
Aripiprazole	34.2% (65)	30.8% (37)	*25.2% (24)*	*52% (13)*
Cyamemazine	8.4% (16)	10% (12)	*11.6% (11)*	*4% (1)*
Olanzapine	4.2% (8)	3.3% (4)	*4.2% (4)*	*0*
Loxapine	1.6% (3)	2.5% (3)	*3.2% (3)*	*0*
Quetiapine	1.1% (2)	0.8% (1)	*1.% (1)*	*0*
**AP generation**				
SGA	91.6% (174)	90% (108)	*88.4% (84)*	*96% (24)*
FGA	8.4% (16)	10% (12)	*11.6% (11)*	*4% (1)*

Legend: AE: adverse event, AP: antipsychotic, N: number of patients, CGI-S: Clinical Global Impression-Severity, CGAS: children’s global assessment scale graduated from 10 − 1 (“needs constant supervision”) to 100 − 91 (“superior functioning”), DSM: Diagnostic and Statistical Manual of Mental Disorder DSM-IV version, SGA: second generation antipsychotic, FGA: first generation antipsychotic

**Table 2:** Types of psychiatric adverse events

Type of psychiatric AEs	All	*AEs Attributable* *to AP*	*AEs* *Non attributable to AP*
**Number of psychiatric AEs** % (n)	100% (374)	*55.9% (209)*	*44.1% (165)*
**Aggressivness/agitation/challenging behaviors** % (n) *	**22.7% (85)**	*15.3% (32)*	*32% (53)*
Agressivness/impulsivity/anger	14.7% (55)	*9% (19)*	*21.8% (36)*
Behavior disorder	1.6% (6)	*0.5% (1)*	*3% (5)*
Motor restlessness	6.4% (24)	*5.7% (12)*	*7.3% (12)*
**Mood changes** % (n) *	**18.4% (69)**	*23.9% (50)*	*11.5% (19)*
Elevated mood	7.5% (28)	*12% (25)*	*1.8% (3)*
Sad or depressed mood	6.4% (24)	*9% (19)*	*3% (5)*
Emotional lability	4.5% (17)	*2.8% (6)*	*6.7% (11)*
**Suicidal ideation/behavior** % (n)	**11.8% (44)**	*12.4% (26)*	*10.9% (18)*
**Apathy/restricted range of emotion/lack of interest** % (n) *	**10.9% (41)**	*15.3% (32)*	*5.4% (9)*
**Irritability** % (n)	6.4% (24)	*5.7% (12)*	*7.3% (12)*
**Trouble paying attention/concentrating** % (n)	7.8% (29)	*9.6% (20)*	*5.5% (9)*
**Anxiety** % (n)	5.6% (21)	*3.8% (8)*	*7.9% (13)*
**Hallucinations** % (n)	3.7% (14)	*2.4% (5)*	*5.5% (9)*
**Racing thoughts** % (n)	2.9% (11)	*1.9% (4)*	*4.2% (7)*
**Sexual dysfonction** % (n)	2.4% (9)	*3.8% (8)*	*0.6% (1)*
**Psychiatric relapse** % (n)	2.4% (9)	*1.9% (4)*	*3% (5)*
**Others (enuresis, encopresis, addiction, scaring, swinging, etc.)** % (n)	4.8% (18)	*3.8% (8)*	*6% (10)*

Legend: AEs = adverse events, *statistically significant difference (p < 0.005) between attributable and non-attributable to AP groups lie to “Aggressiveness/agitation/challenging behaviors”, “Mood changes” and “Apathy/restricted range of emotion/lack of interest” types

Descriptive results of continuous variables are expressed as means (± SD) and as absolute numbers and relative frequencies for categorical variables. The comparison of characteristics between patients with or without at least one psychiatric AE potentially attributable to the AP was performed using the χ² test (or Exact Fisher Test for small sample). Concerning CGI and CGAS scores, the number of AEs and psychiatric AEs were compared using the Kuskall-Wallis test. The incidence rate of psychiatric AEs was evaluated by dividing the number of patients with a new AE during the entire follow-up period by the number of person years at risk. The 95% confidence intervals (CI) are also presented. The comparison of psychiatric AEs according to those different parameters was performed using the χ² test (or Exact Fisher Test for small sample). If necessary, post hoc tests were performed (no alpha risk correction was performed). Statistical analyses were conducted using SAS Enterprise Guide 7.1 (Copyright (c) 2017 by SAS Institute Inc., Cary, NC, USA).

## Results

The characteristics of the ETAPE population are shown in Table 1. The mean age was 12.2 (**±** 3.1) years, with a predominant proportion of boys (70.8%). The main clinical indications for AP treatment in AP-naïve children and adolescents included were “Schizophrenia and other psychotic disorders” (30%), “disruptive, impulse-control, and conduct disorders” (19.2%), “autism spectrum disorder” (10.8%) as well as “personality disorders” (10.8%). We found no statistically significant difference between attributable and non attributable groups for age (*p = 0.40*), sex (*p = 0.39*), pubertal status (*p = 0.73*) and clinical diagnosis (*p = 0,81*).

At the beginning of the study, patients presented a mean CGI-S score of 4.85 (**±** 0.92, a score of 4 “moderate illness” and 5 “marked illness”), consistent with the burden of severe mental illness in the study population. This was also reflected in the measurement of social functioning with a mean CGAS score of 46.2 (**±** 9.9) (score of 50–41 representing a “moderate” and 60 − 51 “variable functioning with sporadic difficulties). No statistically significant difference was found between the different CGI scores and the number of overall psychiatric AEs; and also, between the different CGAS scores and the number of overall psychiatric AEs.

Risperidone and aripiprazole were the most prescribed, respectively, 52.5% and 30.8%, among patients exposed to the same AP molecules throughout the study (n = 125 patients). No statistically significant difference was demonstrated between the molecule AP drug and the distribution of psychiatric AEs (*p = 0.42*). On inclusion, 26% of patients were with comedications (35% anxiolytic, 26% AP, 12% antidepressant, 10% psychostimulant, 10% more than three psychotropics, and 6% thymoregulator treatment).

Within the ETAPE sample of 190 pediatric patients, a total of 2447 AEs were reported. Patients with at least one psychiatric AE were 63% (n = 120). Among the different clinical dimensions of AEs, psychiatric AEs represented 15.28% (374/2447). The overall psychiatric AEs incidence rate was 1.70 per person-year (IC 95% [1.40; 2.01]). Moreover, 55.88% of psychiatric AEs were attributable to SGA by the clinician, compared to 89% of non-psychiatric AEs (*p < 0.001*).

The Table 2 shows the different types of psychiatric AEs observed during the follow-up. For 75% of children and adolescents (n = 95) treated by AP, psychiatric AEs were possibly or probably attributable to AP treatment.

The most frequent observed psychiatric AEs were externalized behaviours such as “Aggressiveness, agitation or challenging behaviours” (22.7%), “Mood changes” (18.4%) and “Suicidal ideas or behaviours” (11.8%). Moreover, we found a statistically significant difference (*p < 0.005*) when comparing the distribution of the types of psychiatric AEs according to imputability to AP drugs. In fact, psychiatric AEs “Mood changes” and “Apathy / restricted range of emotion/lack of interest” were significantly more frequently attributable to AP treatment following the clinical investigator’s judgment (respectively *p = 0.0021* and *p = 0.0025)*, contrary to “Aggressiveness / agitation / challenging behaviors” that were declared significantly less frequently attributed to AP (*p = 0.0001)*.

The severity of the psychiatric AEs is presented in Table 3. Here again, a statistically significant difference in the severity of AEs is exposed according to their imputability to AP. More specifically, there are more mild AEs declared among attributable AEs (post hoc test, *p = 0.0008*), contrary to severe AEs more frequently described in the non attributable group (*p = 0.0001*). There have been five psychiatric AEs of “extreme severity”, three attributable to AP treatment (stabbing, suicidal ideation and behavior), and two non attributable (tantrum and suicide attempt).

**Table 3:** Severity of psychiatric adverse events

Severity	All psychiatrics AEs n = 374	*AEs attributable* *to AP* *n = 209*	*AEs non attributable* *to AP * *n = 165*
N % (n)	100% (374)	*100% (209)*	*100% (165)*
Mild***	49.73% (186)	***57.42% (120)***	*40% (66)*
Moderate	32.35% (121)	*31.58% (66)*	*33.33% (55)*
Severe***	16.58% (62)	*9.57% (20)*	***25.45% (42)***
Extreme	1.34% (5)	*1.44% (3)*	*1.21% (2)*

Legend: AE = adverse event; N = number of AEs; *** = p < 0.001 (significance concerning psychiatric AEs attributable or non attributable to AP)

Table 4 shows the distribution of patients presenting a first psychiatric AE. More than half of patients had a first psychiatric AE (68.3%) during the first quarter (Q1), compared to 19.2% during the second quarter (Q2), 7.5% during the third (Q3), and 5% during the fourth (Q4). No significant difference was found between “Psychiatric AEs attributable to AP” and “Psychiatric AEs non attributable to AP” (*p = 0.6156*).

**Table 4:** The distribution of patients presenting a first psychiatric adverse event

Quarterly follow-up (Q)	All psychiatric AEs (n = 120)	*AEs* *attributable to AP* *(n = 95)*	*AEs* *non attributable to AP* *(n = 25)*
Q1 (n = 167)	68.3% (82)	*70.5% (67)*	*60% (15)*
Q2 (n = 135)	19.2% (23)	*17.8% (17)*	*24% (6)*
Q3 (n = 114)	7.5% (9)	*7.4% (7)*	*8% (2)*
Q4 (n = 108)	5% (6)	*4.2% (4)*	*8% (2)*

Legend : n = number of patients; AE = adverse event; Q = quarter of follow-up; Q1 = 1st to 3rd month; Q2 = 4th to 6th month; Q3 = 7th to 9th month; Q4 = 10th to 12th month of follow-up

The distribution of the occurrence of the different groups of psychiatric AEs during the quarterly follow-up is presented in Supplementary Table S1. There was no evidence of statistically significant difference in the type of psychiatric AE distribution according to the quarter of onset for “the attributable group” and “the non attributable group”, respectively, *p = 0.47* and *p = 0.10*. However, during Q2, the distribution is statistically different according to their imputability (*p = 0.01*): “Mood changes” are more frequent within attributable AEs, contrary to “Aggressivity/agitation/challenging behaviors” and “Anxiety” which are more related to non attributable AEs.

## Discussion

This manuscript describes psychiatric AEs in AP-naïve children and adolescents treated with AP over 12 months during the ETAPE study [26, 28]. The literature reports a large number of AEs attributable to APs, such as clinical AEs (e.g. sedation, extrapyramidal AE or weight gain), and biological AEs (e.g. increased level of prolactin, cholesterol and glucose) [10, 29]. However, few studies examine or mention psychiatric AEs in this young patient population [3, 22, 23]. As shown here, psychiatric AEs can be severe, frequently observed in the pediatric population after being introduced to and during AP treatment (including externalized and internalized AEs), and should therefore be known and correctly identified. We are aware that the natural design of this study cannot delineate whether AP have a direct link with psychiatric AE or whether psychiatric AE are the consequence of non-efficient prescription or both. However, we found it intriguing that this type and severity of psychiatric AEs could influence the clinician’s judgment about its imputability.

Despite a limited list of approvals, APs have taken a central place in the treatment of mental health disorders in the pediatric population. Within APs, risperidone and aripiprazole are the most AP prescribed [5, 30]. The results of our research reflect this use with a strong representation of these two molecules in prescriptions (Table 1). The demographic characteristics of ETAPE population are comparable to those previously reported [22]. In fact, male adolescents are on average 12 years old (SD ± 3.5) years, specifically 13 (SD ± 3.6) years for aripiprazole and 11.6 (SD ± 3.4) years for risperidone, and are more exposed to AP than girls; and particularly to risperidone.

In our prospective naturalistic study, the overall psychiatric AEs incidence rate was 1.70 per person-year, and psychiatric AEs were 15.28% of all AE reported. The very few studies to compare our results to come from pharmacovigilance databases [15, 22]. Moreover, we cannot compare our incidence rate as pharmacovigilance studies are not appropriate to determine an incidence rate. Nevertheless, the proportion of psychiatric AEs we found in ETAPE is in line with previous reports from pharmacovigilance databases. In Rafaniello’s study, which analyzed spontaneously reported AEs among children and adolescents treated with aripiprazole or risperidone using the EudraVigilance database from 2016 to 2018 [22], the rate of psychiatric AEs was 20.2% (with suicidal behavior reported in 14.9%) on aripiprazole and 15% on risperidone. The Minjon’s study, which analyzed AEs reported under AP (mainly risperidone, aripiprazole and quetiapine) in children ages 1–17 years from the global VigiBase database, found a rate of psychiatric AEs of 13.2% [15].

In our pediatric population, three types of psychiatrics AEs were the most represented with “Aggressiveness, agitation or challenging behaviors”, “Mood changes”, and “Suicidal ideas or behaviors”, (representing respectively 22,7%, 18,4% and 11,8% of psychiatric AEs) (Table 2).

We didn’t show any association between diagnoses (Diagnostic and Statistical Manual), disease severity (CGI) or social functioning (CGAS), sex and the presence of psychiatric AEs (*p > 0.05*); but some studies made other observations [3, 15]. Jakobsen and al reported interesting results concerning aripiprazole-associated psychiatric events in children and adolescents through the database of the Danish Medicines Agency. In patients with psychotic disorders, aripiprazole could lead to aggressive behavior, anxiety, hallucinations, mental tics, neuroleptic malignant syndrome, overeating, and suicidal behavior [3]. In the study of Minjon et al., depression, suicide/self-injury, drug abuse, dependence, and withdrawal were less frequently reported in males than in females; in contrary to hostility/aggression more frequently reported in males. Moreover, depression and suicide/self-injury, drug abuse, dependence, and withdrawal were relatively less frequently reported in children ages 1–11 than in children ages 12–17. But hostility/aggression were relatively more frequently reported in children ages 6–11 than in children ages 12–17. In addition, AEs were more frequently reported by health care professionals compared with consumers [15].

On the other hand, in ETAPE study, we observed that psychiatric AEs are significantly less considered to be “related” to the AP drug than non-psychiatric AEs (55.88% against 89%). Likewise, some internalized AEs (such as mood change or negative symptoms) are attributed to AP drug by the on-site investigator; however, the externalized symptoms (as “Aggressiveness, agitation or challenging behaviours” type) are less attributed to the AP drug. This may suggest that they are related to the underlying mental health disorders; whereas these symptoms were not present before the introduction of the AP. These observations raise the issue of whether the type of AE might influence the clinician’s judgment about imputability to the AP molecule [27]. In the same way, the most severe psychiatric AEs are non attributable to the molecule AP, which may also suggest that they are linked to mental disorders (Table 3).

Regarding mood swings, well-designed clinical trials, carried out in the adult population, suggest that AP-induced mania/hypomania is a marginal phenomenon [31, 32].

Suicidal behaviors as AEs during AP treatment as observed in our pediatric study population, have also been reported by other authors [22, 23, 33]. Kimura et al., 2015, analyzed reports submitted to the United States Food and Drug Administration (FDA) Adverse Event Reporting System (FAERS) from 1997 to 2011 to assess serious AEs induced by the administration of APs to children aged 0 to 12 years. Signals in the data that signified a drug-associated AE were detected via quantitative data mining algorithms. Signal scores for AP-associated suicide have been reported with a statistically significant association with haloperidol, olanzapine, quetiapine, risperidone and aripiprazole. The signal scores were higher for olanzapine and risperidone [23].

Nevertheless, even if some evidence points to psychiatric AEs, including suicidal behaviors in AP-treated pediatric populations presenting mental health disorders, more specific studies, including control groups, are required to verify the imputability of AP treatment for those AEs [22]. The interpretation of the possible relationship between AP use and suicidal behavior is not clarified, in contrast with antidepressants for which placebo-controlled trials and meta-analysis have reported a moderate increase of suicidal behavior [34]. Indeed, it is difficult to know if these behaviors are induced by the AP medication or caused by the mental illness for which the treatment is prescribed. Suicidal behavior is highly associated with psychiatric conditions, particularly mood and psychotic disorders [35, 36]. What we know today, among the APs, clozapine was the only AP which has been associated with decreased risk of attempted or completed suicide in the Swedish cohort included all persons aged 16–64 with schizophrenia diagnoses [37, 38].

Concerning the onset kinetics of the first AEs in the pediatric population (Table 4), more than half of the patients (68.3%) from Q1 had a first psychiatric AE. This observation is in line with literature data which shows that AEs under AP occurred within 3 months after taking the medicine [39]. But the Table 4 provides an additional finding, as psychiatric AEs continue to appear beyond Q1.

Moreover, it is also necessary to underline the risk of polypharmacy which makes it challenging to understand the occurrence of AEs and can lead to severe AEs [33, 40].

## Limitations of the study

In ETAPE study, we have noted these limitations: (1) the small number of patients in the sub-groups does not allow generalizing the results; (2) all categories of AEs were systematically screened using the PAERS, which may have led to higher detection of AEs compared to studies with spontaneous reporting; (3) psychiatric AEs were not specifically researched; (4) there was no control group and (5) causality assessment was completely dependent on expert judgments.

## Conclusion

ETAPE results show that psychiatric AEs are observed in pediatric patients treated by AP. Thus, clinicians need to be aware of the possibility of occurrence of psychiatric AEs while prescribing APs. Furthermore, the relation to AP treatment should systematically be assessed and monitored for any novel or worsening psychiatric symptoms, also considering the evolution of the underlying psychiatric condition. The risk of psychiatric AEs should be part of the benefit-risk revaluation when prescribing an AP to pediatric patients. AEs should be monitored, especially during the first months after introducing an AP drug to this population. Further studies are needed in this area on larger samples to better understand the occurrence of psychiatric AEs, with the mechanisms underlying them and the role of AP drug treatment.

## Electronic Supplementary Material

Below is the link to the electronic supplementary material.

Supplementary Material 1: Table S1: Distribution of psychiatric Adverse Events during the quarterly follow-up

## Data Availability

The database is available.

## Abbreviations

AP: Antipsychotics
AEs: Adverse events
AACAP: American Academy Child and Adolescent Psychiatry
ANSM: The French National Agency for Medicines and Health Products Safety
CAMESA: Canadian Alliance for Monitoring Effectiveness and Safety of Antipsychotics in Children
CGI-S: Clinical Global Impressions-Scale
CGAS: Children Global Assessment Scale
CI: Confidence Intervals
FAERS: FDA Adverse Event Reporting System
FDA: Food and Drug Administration
NICE: National Institute for Health and Care Excellence
PAERS: Pediatric Adverse Events Rating Scale
Q: Quarter
SD: Standard Deviations

## References

[1] Bushnell GA, Crystal S, Olfson M (2021). Trends in Antipsychotic Medication Use in Young Privately Insured Children. J Am Acad Child Adolesc Psychiatry.

[2] Lee ES, Vidal C, Findling RL (2018). A Focused Review on the Treatment of Pediatric Patients with Atypical Antipsychotics. Child Adolesc Psychopharmacol.

[3] Jakobsen KD, Bruhn CH, Pagsberg AK, Fink-Jensen A, Nielsen J (2016). Neurological, metabolic, and psychiatric adverse events in children and adolescents treated with aripiprazole. J Clin Psychopharmacol.

[4] Olfson M, Crystal S, Huang C, Gerhard T (2010). Trends in antipsychotic drug use by very young, privately insured children. J Am Acad Child Adolesc Psychiatry.

[5] Halfdanarson O, Zoega H, Aagaard L (2017). International trends in antipsychotic use: A study in 16 countries, 2005–2014. Eur Neuropsychopharmacol.

[6] Vitiello B, Correll C, van Zwieten-Boot B, Zuddas A, Parellada M, Arango C (2009). Antipsychotics in children and adolescents: increasing use, evidence for efficacy and safety concerns. Eur Neuropsychopharmacol.

[7] Kaguelidou F, Holstiege J, Schink T, Bezemer I, Poluzzi E, Mazzaglia G, Pedersen L, Sturkenboom M, Trifirò G (2020). Use of antipsychotics in children and adolescents: a picture from the ARITMO population-based European cohort study. Epidemiol Psychiatr Sci.

[8] Benard-Laribiere A, Noize P, Girodet PO, Lassalle R, Dureau-Pournin C, Droz-Perroteau C, Fourrier-Reglat A, Salvo F, Bezin J, Pariente A, DRUGS-2M Study Group (2019). Monitoring of drug misuse or potential misuse in a nationwide healthcare insurance database: A cross-sectional study in France. Therapie.

[9] Onishi Y, Mikami K, Kimoto K, Watanabe N, Takahashi Y, Akama F, Yamamoto K, Matsumoto H (2021). Second-generation antipsychotic drugs for children and adolescents. J Nippon Med Sch.

[10] Krause M, Zhu Y, Huhn M, Schneider-Thoma J, Bighelli I, Chaimani A, Leucht S (2018). Efficacy, acceptability, and tolerability of antipsychotics in children and adolescents with schizophrenia: A network meta-analysis. Neuropsychopharmacol.

[11] Cohen D, Bonnot O, Bodeau N, Consoli A, Laurent C (2012). Adverse effects of second-generation antipsychotics in children and adolescents: a Bayesian meta-analysis. J Clin Psychopharmacol.

[12] Fraguas D, Correll CU, Merchán-Naranjo J, Rapado-Castro M, Parellada M, Moreno C, Arango C (2011). Efficacy and safety of second-generation antipsychotics in children and adolescents with psychotic and bipolar spectrum disorders: comprehensive review of prospective head-to-head and placebo-controlled comparisons. Eur Neuropsychopharmacol.

[13] Zuddas A, Zanni R, Usala T (2011). Second generation antipsychotics (SGAs) for non-psychotic disorders in children and adolescents: a review of the randomized controlled studies. Eur Neuropsychopharmacol.

[14] Correll CU (2008). Assessing and maximizing the safety and tolerability of antipsychotics used in the treatment of children and adolescents. J Clin Psychiatry.

[15] Minjon L, Aarts JW, van den Ban E, Cg Egberts T, Heerdink ER: Clarity and applicability of adverse drug reaction-related monitoring instructions in clinical practice guidelines for children and adolescents treated with antipsychotic drugs: A review of six clinical practice guidelines. Review BMJ Open. 2022;12(3):e058940. 10.1136/bmjopen-2021-058940 PMC8905889 35260462

[16] Raffin M, Giannitelli M, Consoli A, Bonnot O, Menard ML, Askenazy F, Laurent C, Cohen D (2014). Management of adverse effects of second-generation antipsychotics in youth. Cur Treat Opt Psychiatry.

[17] Kendall T, Hollis C, Stafford M, Taylor C, Guideline Development Group (2013). Recognition and management of psychosis and schizophrenia in children and young people: summary of NICE guidance. BMJ.

[18] Ho J, Panagiotopoulos C, McCrindle B, Grisaru S, Pringsheim T (2011). CAMESA guideline group: Management recommendations for metabolic complications associated with second-generation antipsychotic use in children and youth. J Can Acad Child Adolesc Psychiatry.

[19] Jazi S, Ben Amor L, Abadie P, Menard ML, Berthiaume C, Mottron L, Choquette R, Ilies D (2021). Long-term metabolic monitoring of youth treated with second-generation antipsychotics five years after the CAMESA guidelines: are we making progress?. Can J Psychiatry.

[20] Dinnissen M, Dietrich A, van der Molen JH, Verhallen AM, Buiteveld Y, Jongejan S, Troost PW, Buitelaar JK, Hoekstra PJ, van den Hoofdakker BJ (2020). Prescribing antipsychotics in child and adolescent psychiatry: guideline adherence. Eur Child Adolesc Psychiatry.

[21] Jeon SM, Park S, Kwon S, Kwon JW (2021). Association Between Antipsychotic Treatment and Neurological Adverse Events in Pediatric Patients: A Population-Based Cohort Study in Korea. Front Psychiatry.

[22] Rafaniello C, Sullo MG, Carnovale C, Pozzi M, Stelitano B, Radice S, Bernardini R, Rossi F, Clementi E, Capuano A (2020). We Really Need Clear Guidelines and Recommendations for Safer and Proper Use of Aripiprazole and Risperidone in a Pediatric Population: Real-World Analysis of EudraVigilance Database. Front Psychiatry.

[23] Kimura G, Kadoyama K, Brown JB, Nakamura T, Miki I, Nisiguchi K, Sakaeda T, Okuno Y (2015). Antipsychotics-associated serious adverse events in children: An analysis of the FAERS database. Int J Med Sci.

[24] Aagaard L, Hansen EH (2010). Adverse drug reactions from psychotropic medicines in the paediatric population: analysis of reports to the Danish Medicines Agency over a decade. BMC Res Notes.

[25] https://www.ema.europa.eu/en/ich-e2a-clinical-safety-data-management-definitions-standards-expedited-reporting.

[26] Menard ML, Thümmler S, Giannitelli M, Olliac B, Bonnot O, Cohen D, Askenazy F, ETAPE Study group (2016). Incidence of adverse events in naïve children and adolescents treated with antipsychotic drugs: a French multicenter naturalistic study protocol (ETAPE). BMJ Open.

[27] Purushotham Naid R (2013). Causality assessment: A brief insight into practices in pharmaceutical industry. Perspect Clin Res.

[28] Menard ML, Thümmler S, Giannitelli M, Roger C, Bonnot O, Cohen D, Askenazy F (2019). ETAPE Study group: Incidence of adverse events in naive children and adolescents treated with antipsychotic drugs. Eur Neuropsychopharmacol.

[29] Solmi M, Fornaro M, Ostinelli EG, Zangani C, Croatto G, Monaco F, Krinitski D, Fusar-Poli P, Correll CU (2020). Safety of 80 antidepressants, antipsychotics, anti-attention-deficit/hyperactivity medications and mood stabilizers in children and adolescents with psychiatric disorders: a large scale systematic meta-review of 78 adverse effects. World Psychiatry.

[30] Minjon L, van den Ban E, de Jong E, Souverein PC, Egberts TCG, Heerdink ER (2019). Reported adverse drug reactions in children and adolescents treated with antipsychotics. J Child Adolesc Psychopharmacol.

[31] Benyamina A, Samalin L (2012). Atypical antipsychotic-induced mania/hypomania: a review of recent case reports and clinical studies. Rev Int J Psychiatry Clin Pract.

[32] Rachid F, Bertschy G, Bondolfi G, Aubry JM (2004). Possible induction of mania or hypomania by atypical antipsychotics: an updated review of reported cases. Rev J Clin Psychiatry.

[33] Coustals N, Menard ML, Cohen D (2020). Aripiprazole in children and adolescents. J Child Adolesc Psychopharmacol.

[34] Cipriani A, Zhou X, Del Giovane C (2016). Comparative efficacy and tolerability of antidepressants for major depressive disorder in children and adolescents: a network meta-analysis. Lancet.

[35] Brådvik L. Suicide risk and mental disorders: Int J Environ Res Public Health. 2018; 15(9): 2028. 10.3390/ijerph15092028 PMC6165520 30227658

[36] Pompili M, Baldessarini RJ, Forte A, Erbuto D, Serafini G, Fiorillo A, Amore M, Girardi P (2016). Do atypical antipsychotics have antisuicidal effects? A hypothesis-generating overview. Rev Int J Mol Sci.

[37] https://www.fda.gov/safety/reporting-serious-problems-fda/how-consumers-can-report-adverse-event-or-serious-problem-fda.

[38] Taipale H, Lähteenvuo M, Tanskanen A, Mittendorfer-Rutz E, Tiihonen J (2021). Comparative effectiveness of antipsychotics for risk of attempted or completed suicide among persons with schizophrenia. Schizophr Bull.

[39] Guo K, Feng Z, Chen S, Yan Z, Jiao Z, Feng D (2022). Safety profile of antipsychotic drugs: analysis based on a provincial spontaneous reporting systems Database. Front Pharmacol.

[40] Jeon SM, Park S, Kim D, Kwon JW (2021). Risk of seizures associated with antipsychotic treatment in pediatrics with psychiatric disorders: a nested case-control study in Korea. Eur Child Adolesc Psychiatry.

