# Publisher Correction: Emergence of psychiatric adverse events during antipsychotic treatment in AP-naïve children and adolescents

**DOI:** 10.1186/s13034-023-00580-4

**Published:** 2023-03-10

**Authors:** Marie-Line Menard, Philippe Auby, Coralie Cruzel, David Cohen, Olivier Bonnot, Florence Askenazy, Susanne Thümmler, ETAPE Study Group, Boublil Michel, Castaings Agnès, Catanese Alexandre, Chambry Jean, Charvet Dorothée, Cseterky Mona, Fernandez Arnaud, Fontas Eric, Fourneret Pierre, Giannitelli Marianna, Gicquel Ludovic, Kabuth Bernard, Leroy Bernard, Maria Fanny, Moceri Pamela, Olliac Bertrand, Raynaud Jean-Philippe, Roche Jean-François, Rochet Thierry

**Affiliations:** 1 Department of Child and Adolescent Psychiatry, University, Children’s Hospitals of Nice CHU- Lenval, 57 Avenue de La Californie, 06200 Nice, France; 2 CoBTek, EA7276, Université Côte d’Azur, Nice, France; 3 Department of Clinical Research and Innovation (DRCI), Nice University Hospital, Nice, France; 4 Department of Child and Adolescent Psychiatry, GH Pitié-Salpêtrière, APHP, Paris, France; 5 CNRS UMR 7222 Institut des Systèmes Intelligents et Robotiques, Pierre & Marie Curie University, Paris, France; 6 Department of Child and Adolescent Psychiatry, University Hospital, Nantes, France

## Publisher Correction: Child and Adolescent Psychiatry and Mental Health (2022) 16:83 https://doi.org/10.1186/s13034-022-00517-3

Following publication of the original article [1], one of the collaborators (Fernandez Arnaud) of ETAPE Study Group has noticed that the collaborator names are not referenced as a collaborator on PubMed. The list of collaborators is present at the end of the original article under the section "Collaborators ETAPE study group" but not referenced in Pubmed due to the XML tagging error. Therefore, this has been corrected with this erratum.

The original article has been corrected.
