# Supplementary material for: Emergence of psychiatric adverse events during antipsychotic treatment in AP-naïve children and adolescents
Source: Child Adolesc Psychiatry Ment Health. 2022 Nov 12;16:83. doi: 10.1186/s13034-022-00517-3 (PMC9655798; doi:10.1186/s13034-022-00517-3)
Supplement: Supplementary file 1 — Supplementary Material 1: Table S1: Distribution of psychiatric Adverse Events during the quarterly follow-up [file 13034_2022_517_MOESM1_ESM.pdf]

Table S1: Distribution of psychiatric Adverse Events during the quarterly follow-up

	All types of psychiatrics AEs (n=374)				Psychiatric AEs attributable to AP (n=209)				Psychiatric AEs non attributable to AP (n=165)			
**Quarterly follow-up (Q)**	Q1	Q2	Q3	Q4	Q1	Q2	Q3	Q4	Q1	Q2	Q3	Q4
**Number of psychiatric AE % *(n)***	100% (185)	100% (89)	100% (59)	100% (41)	100% (132)	100% (38)	100% (24)	100% (15)	100% (53)	100% (51)	100% (35)	100% (26)
**Aggressiveness/agitation/ challenging behaviors**	20.5% (38)	15.7% (14)	32.2% (19)	34.1% (14)	16.7% (22)	**5.3% (2)****	20.8% (5)	20% (3)	30.3% (16)	**23.5% (12)****	40% (14)	42.3% (11)
**Mood changes**	18.9% (37)	20.3% (18)	13.6% (8)	14.6% (6)	22% (29)	**31.6% (12)****	25% (6)	20% (3)	15.1% (8)	**11.7% (6)****	5.8% (2)	11.7% (3)
Suicidal ideation/behavior	10.8% (20)	15.7% (14)	11.9% (7)	7.3% (3)	11.4% (15)	18.4% (7)	12.5% (3)	6.7% (1)	9.4% (5)	13.7% (7)	11.4% (4)	7.7% (2)
Apathy/restricted range of emotion/lack of interest	11.3% (21)	11.1% (10)	6.8% (4)	14.6% (6)	13.7% (18)	15.7% (6)	12.5% (3)	33.3% (5)	5.7% (3)	7.8% (4)	2.9% (1)	3.9% (1)
Irritability	9.2% (17)	1.1% (1)	6.8% (4)	4.9% (2)	7.6% (10)	0	0	13.3% (2)	13.2% (7)	2% (1)	11.4% (4)	0
Trouble paying attention/concentrating	9.2% (17)	9% (8)	6.8% (4)	0	10.6% (14)	13.2% (5)	4.2% (1)	0	5.7% (3)	5.9% (3)	8.6% (3)	0
**Anxiety**	3.8% (7)	12.4% (11)	3.4% (2)	2.4% (1)	4.5% (6)	**2.6% (1)****	4.2% (1)	0	1.9% (1)	**19.6% (10)****	2.9% (1)	3.9% (1)
Hallucinations	4.3% (8)	3.4% (3)	1.7% (1)	4.9% (2)	3% (4)	2.6% (1)	0	0	7.6% (4)	3.9% (2)	2.9% (1)	7.7% (2)
Racing Thoughts	2.2% (4)	3.4% (3)	3.4% (2)	4.9% (2)	1.5% (2)	0	8.3% (2)	0	3.8% (2)	5.9% (3)	0	7.7% (2)
Sexual dysfunction	3.2% (6)	2.2% (2)	1.7% (1)	0	3.8% (5)	5.3% (2)	4.2% (1)	0	1.9% (1)	0	0	0
Psychiatric relapse	2.7% (5)	1.1% (1)	0	7.3% (3)	2.3% (3)	0	0	6.7% (1)	3.8% (2)	2% (1)	0	7.7% (2)
Others	2.7% (5)	4.5% (4)	11.8% (7)	4.9% (2)	3% (4)	5.3% (2)	8.3% (2)	0	1.9% (1)	1.9% (1)	8.6% (3)	7.7% (2)

Legend: AE = Adverse Event; Q = quarter of follow-up; Q1 = 1st to 3rd month; Q2 = 4th to 6th month; Q3 = 7th to 9th month; Q4 = 10th to 12th month of follow-up. **During the 2nd quarter of follow-up (Q2), the distribution of psychiatric AEs is statistically different according to their imputability ($p<0.01$). The differences concerning attributability of AEs during Q2 are significant for the following psychiatric AEs: "Aggressiveness/agitation/challenging behaviors" and "Anxiety" (with more AEs of this type among not attributed AEs), as well as "Mood changes" (with more AEs of this type among attributable AEs).
